# Cervical Cytology and HPV16/18/45 mRNA Co-Testing Improve Risk Stratification in Routine Clinical Practice

**DOI:** 10.3390/cancers18050834

**Published:** 2026-03-04

**Authors:** Sveinung Wergeland Sørbye, Bente Marie Falang, Mona Antonsen, Elin Richardsen

**Affiliations:** 1 Department of Clinical Pathology, University Hospital of North Norway, 9006 Tromsø, Norway; mona.antonsen@unn.no (M.A.); elin.richardsen@unn.no (E.R.); 2 PreTect AS, 3490 Klokkarstua, Norway; bente.falang@pretect.no

**Keywords:** cervical cancer screening, HPV *E6/E7* mRNA, PreTect SEE, genotype-specific testing, HPV mRNA triage, NILM, ASC-US, CIN2+, CIN3+, cervical cancer, real-world cohort, Norway

## Abstract

**Background/Objectives:** Co-testing may improve cervical cancer prevention by stratifying women into groups with different absolute risks of CIN2+, CIN3+, and cervical cancer. We evaluated real-world co-testing with cervical cytology and a genotype-specific HPV *E6/E7* mRNA assay targeting HPV16, HPV18, and HPV45 (PreTect SEE) in routine clinical practice. **Methods:** We conducted a retrospective, registry-based cohort study at the Department of Clinical Pathology, University Hospital of North Norway. Eligible co-test samples (liquid-based cytology with concurrent HPV mRNA testing, both with valid results) from routine screening, follow-up, and clinically indicated testing were identified from the laboratory information system and passively followed for worst histological outcome through December 2025. Outcomes were no biopsy/<CIN2, CIN2+, CIN3+, and cervical cancer. **Results:** Among 116,217 eligible co-test samples (mean age 43.9 years), cumulative risks were 4.4% for CIN2+, 1.5% for CIN3+, and 0.1% for cervical cancer. Baseline HPV mRNA positivity was 3.9%, and cytology was ASC-US+ in 12.2% of samples. Co-testing produced a clear stepwise risk gradient. Double-negative results (NILM/mRNA−; 86.7%) had very low risks (CIN3+ 0.2%; cervical cancer 0.02%). ASC-US+/mRNA− results (9.3%) showed intermediate risks (CIN3+ 4.1%; cervical cancer 0.2%). NILM/mRNA+ results (1.1%) showed substantially higher risks despite normal cytology (CIN3+ 13.0%; cervical cancer 0.5%). Double-positive results (ASC-US+/mRNA+; 2.8%) had the highest risks (CIN3+ 28.5%; cervical cancer 2.3%). Within NILM, mRNA positivity captured 42.7% of CIN3+ cases and 25.0% of cancers. Genotype-specific analyses showed highest risks for HPV16, followed by HPV18 and HPV45. **Conclusions:** Co-testing with cervical cytology and a 3-type HPV mRNA assay provided strong, clinically interpretable risk stratification and identified a small but high-risk subgroup among women with normal cytology. These findings support genotype-specific HPV mRNA testing as an adjunct to cytology in routine care.

## Simple Summary

We evaluated whether adding a targeted HPV mRNA test to routine Pap testing could better identify women at increased risk of serious cervical precancer or cervical cancer in everyday clinical practice. The test detects activity from three HPV types (16, 18, and 45) strongly linked to cervical cancer. Using routine data from Northern Norway, we analyzed more than 116,000 combined Pap and HPV mRNA tests and linked them to later biopsy outcomes. Combined testing separated women into clear risk groups: risk was very low when both tests were negative and increased when one or both tests were positive. Women with a normal Pap test but a positive HPV mRNA test had a clearly higher later risk of cervical cancer than women with both tests negative, identifying a small but clinically important risk group. These findings support HPV mRNA testing as an adjunct to cytology for risk-based follow-up.

## 1. Introduction

Persistent infection with oncogenic human papillomavirus (HPV) is a necessary cause of cervical cancer [1,2], and organized screening programs have substantially reduced cervical cancer incidence and mortality through detection and treatment of high-grade precursor lesions [3,4]. Many countries have transitioned from cytology-based screening to primary HPV DNA testing because of its high sensitivity for cervical precancer [5,6]. However, HPV DNA testing detects both transient and transforming infections, which may increase referral rates and overmanagement unless effective triage strategies are applied [7,8].

Cervical cytology remains widely used either as a primary screening test or as triage following a positive HPV DNA result [9,10]. Nevertheless, cytology has limited sensitivity and substantial interobserver variability, particularly for low-grade abnormalities [11,12]. While cytology–HPV DNA co-testing has shown limited benefit over HPV DNA primary screening, large, randomized trials have demonstrated that HPV DNA-based primary screening provides superior sensitivity, leading most screening guidelines to recommend HPV primary screening without concurrent cytology [13,14]. Importantly, this evidence is based on co-testing strategies combining cytology with broad-spectrum (typically 14-type) HPV DNA assays, where cytology adds limited incremental value beyond HPV DNA alone [15,16]. These findings do not address the potential role of biologically distinct biomarkers, such as genotype-specific HPV *E6/E7* mRNA, which reflect viral oncogene expression rather than viral presence and may therefore provide complementary risk information to cytology and improve risk stratification [17,18].

HPV *E6/E7* mRNA testing is biologically closer to malignant transformation than DNA detection, as it targets viral oncogene expression rather than viral presence alone [17,19]. Consequently, HPV mRNA assays typically demonstrate lower test positivity and higher clinical specificity than HPV DNA tests in triage settings, with the potential to reduce unnecessary colposcopy and treatment while maintaining strong discrimination for CIN3+ [20,21,22]. The 3-type HPV mRNA assay PreTect SEE detects and genotypes *E6/E7* mRNA from HPV16, HPV18, and HPV45—genotypes that contribute disproportionately to cervical cancer, particularly adenocarcinoma—making it a candidate tool for identifying a small subset of women at particularly high risk, including among those with negative cytology [23].

The Norwegian Cervical Cancer Screening Program was established in 1995 and was based on cervical cytology every 3 years for women aged 25–69 years. Primary HPV DNA testing was introduced gradually from 2019, initially as a 50/50 randomized implementation for women aged 34–69 years (cytology every 3 years versus HPV DNA testing every 5 years), with full implementation in this age group in 2022. From 2023, primary HPV DNA testing every 5 years was recommended for all women aged 25–69 years. During most of the period covered by the present study, however, women were screened according to the cytology-based national program. In this setting, a 3-type HPV mRNA test (PreTect SEE; HPV16/18/45) was added locally as a co-test to cervical cytology to reduce the risk of cervical cancer after normal cytology and to improve risk stratification.

At the Department of Clinical Pathology, University Hospital of North Norway (UNN), cervical samples obtained in routine clinical practice were co-tested with liquid-based cytology and HPV mRNA during defined implementation periods. This created a large real-world cohort with long-term passive follow-up through the laboratory information system, allowing linkage of baseline co-test results to subsequent histological outcomes captured in routine care. Such a setting enables estimation of absolute risks that are directly interpretable in a population-based screening context and relevant for clinical decision-making [24]. Here, “co-testing” refers to cervical cytology plus the 3-type HPV mRNA assay (PreTect SEE; HPV16/18/45), not universal HPV DNA co-testing.

We evaluated co-testing with cervical cytology and a 3-type HPV mRNA assay in an organized cervical cancer screening setting in Northern Norway. Specifically, we (i) described the distribution of histological outcomes according to baseline cytology and HPV mRNA results; (ii) estimated absolute risks of CIN2+, CIN3+, and cervical cancer across four baseline co-test categories (double-negative, cytology-positive only, mRNA-positive only, and double-positive); and (iii) assessed the triage value of HPV mRNA within NILM and ASC-US+ cytology, including the proportion of CIN3+ lesions and cervical cancers captured among mRNA-positive women. We also report outcome risks by genotype-specific HPV mRNA positivity (HPV16, HPV18, and HPV45) to illustrate how oncogene expression patterns align with clinically relevant risk.

## 2. Materials and Methods

### 2.1. Study Design and Setting

We conducted a retrospective, registry-based cohort study within the organized Norwegian cervical cancer screening programme in Northern Norway. The study was carried out at the Department of Clinical Pathology, University Hospital of North Norway (UNN), which serves as the regional pathology provider for Troms and Finnmark counties. UNN processes approximately 25,000 cervical cytology samples annually, encompassing routine screening, follow-up examinations, and samples collected from women evaluated due to clinical symptoms.

Following pilot evaluations of HPV mRNA testing conducted in 2013 and 2014, routine co-testing with liquid-based cytology and a 3-type HPV *E6/E7* mRNA assay targeting HPV16, HPV18, and HPV45 (PreTect SEE) was implemented for all cervical samples processed at UNN during the period 2016–2020 [24,25]. All eligible co-test samples, defined as cervical liquid-based cytology specimens with concurrent HPV mRNA testing and valid results for both assays, analysed during the study period, were included as the unit of analysis. No de-duplication at the individual level was performed. Thus, women could contribute more than one eligible co-test sample across screening rounds and follow-up, and the unit of analysis was the co-test sample rather than the individual.

### 2.2. Data Sources and Study Population

All data were extracted from the laboratory information system SymPathy v5.14.4.2 (Tieto, Helsinki, Finland), used at UNN. SymPathy contains comprehensive local records of cervical cytology, HPV testing, cervical biopsies, loop electrosurgical excision procedure (LEEP)/conization specimens, and hysterectomy specimens from the defined catchment area.

Eligible co-test samples identified from routine clinical practice were linked deterministically within SymPathy using the unique national personal identification number assigned to each individual. Prior to analysis, all data were de-identified to ensure patient confidentiality.

### 2.3. Cervical Cytology

Cervical specimens were collected in ThinPrep^®^ PreservCyt^®^ solution (ThinPrep Pap Test; Hologic Inc., Marlborough, MA, USA) in accordance with routine clinical practice. Samples were processed on the ThinPrep platform and interpreted by certified cytotechnologists and pathologists at UNN using the 2014 Bethesda System for Reporting Cervical Cytology.

For risk stratification analyses, cytology results were grouped into two categories: NILM (negative for intraepithelial lesion or malignancy) and ASC-US+ (atypical squamous cells of undetermined significance or more severe abnormalities, including LSIL, ASC-H, HSIL, adenocarcinoma in situ (AIS), and cytological cancer). In addition, a higher-grade cytology category, ASC-H+ (defined as ASC-H or more severe abnormalities), was reported descriptively.

### 2.4. HPV mRNA Testing (PreTect SEE)

Residual material from the same ThinPrep^®^ PreservCyt^®^ vial was analysed using the 3-type HPV *E6/E7* mRNA assay PreTect SEE (PreTect AS, Klokkarstua, Norway), which targets HPV genotypes 16, 18, and 45. The assay is a qualitative, genotype-specific test based on real-time nucleic acid sequence-based amplification (NASBA), an isothermal RNA amplification method performed at 41 °C, with molecular beacon probes enabling type-specific detection.

Total nucleic acids were isolated from 1 mL of residual PreservCyt material in accordance with the manufacturer’s instructions. Each analytical run included type-specific positive and negative controls, as well as an intrinsic sample control (ISC) targeting a human housekeeping transcript to verify specimen adequacy and to detect potential amplification inhibition. HPV mRNA testing was performed at PreTect AS under routine laboratory conditions and in accordance with the manufacturer’s validated procedures.

### 2.5. Eligibility Criteria and Co-Test Categories

Co-test samples were considered eligible if cervical cytology was satisfactory for evaluation and HPV mRNA testing yielded a valid intrinsic sample control (ISC). Samples were excluded if cytology was unsatisfactory or indeterminate, if personal identifiers were missing and precluded deterministic linkage, if residual material was insufficient for HPV mRNA testing, or if the ISC result was invalid. In cases where repeat sampling occurred following an invalid or unsatisfactory result, the first subsequent co-test sample meeting the eligibility criteria was included in the analysis.

Baseline co-test results were classified into four categories according to cytology and HPV mRNA status:

1. NILM/mRNA− (normal cytology with negative HPV mRNA)
2. ASC-US+/mRNA− (abnormal cytology, defined as ASC-US or worse, with negative HPV mRNA)
3. NILM/mRNA+ (normal cytology with positive HPV mRNA)
4. ASC-US+/mRNA+ (abnormal cytology, defined as ASC-US or worse, with positive HPV mRNA).

For genotype-specific analyses, HPV16, HPV18, and HPV45 mRNA positivity indicators were treated as non-mutually exclusive, allowing a single sample to be positive for more than one genotype.

### 2.6. Follow-Up and Outcome Ascertainment

Women were followed passively through the laboratory information system SymPathy for subsequent histological outcomes recorded in routine clinical care, including cervical biopsies, loop electrosurgical excision procedure (LEEP)/conization specimens, and hysterectomy specimens. Follow-up time was calculated from the date of the index co-test to the earliest occurrence of a relevant histological endpoint, hysterectomy, death, emigration or out-migration from the catchment area, or the end of the observation period. Complete histological follow-up was available through December 2025.

### 2.7. Outcome Definitions

Histological outcomes were defined according to the most severe (worst) histological diagnosis recorded during follow-up. Outcomes were grouped into four categories:

1. No biopsy or <CIN2 (no histological sampling performed, benign/normal histology, or cervical intraepithelial neoplasia grade 1 (CIN1))
2. CIN2+ (CIN2, CIN3, adenocarcinoma in situ (AIS), or invasive cervical cancer)
3. CIN3+ (CIN3, AIS, or invasive cervical cancer)
4. Cervical cancer (invasive squamous cell carcinoma or invasive adenocarcinoma of the cervix).

When both diagnostic biopsy and treatment histology (e.g., LEEP/conization or hysterectomy specimens) were available, the most severe diagnosis was used as the study endpoint. In diagnostically challenging cases, p16(INK4a) immunohistochemistry was used as an adjunct according to routine practice, and discrepant interpretations were resolved by consensus, applying the more severe diagnosis.

### 2.8. Statistical Analysis

Analyses were conducted at the co-test sample level and were primarily based on cross-tabulations of baseline test categories versus the worst histological outcome observed during follow-up. Outcomes were defined as CIN2+, CIN3+ (including adenocarcinoma in situ (AIS) and cervical cancer), and cervical cancer (invasive squamous cell carcinoma or invasive adenocarcinoma).

Absolute risks were reported as cumulative proportions with corresponding numerators/denominators. For key risk estimates and selected clinically relevant contrasts, 95% confidence intervals (CIs) were calculated. Effect sizes were reported as risk ratios (RRs; mRNA-positive vs. mRNA-negative) and absolute risk differences (percentage-point differences) with 95% CIs.

Overall differences in outcome proportions across mutually exclusive groups were evaluated using Pearson’s chi-square tests. A two-sided *p*-value < 0.05 was considered statistically significant. For cervical cancer and other analyses with small numbers of events, statistical significance was interpreted cautiously, and emphasis was placed on effect sizes and absolute risks. Analyses based on nested outcome categories were presented descriptively.

Because genotype-specific HPV mRNA markers (HPV16, HPV18, and HPV45) could co-occur within the same sample, genotype-specific results were interpreted as marker-specific risk associations rather than independent between-group comparisons. To address marker co-expression, an additional analysis among HPV mRNA-positive samples used mutually exclusive HPV16/18/45 genotype-combination groups.

All analyses were performed using IBM SPSS Statistics version 29.0.1.0 (IBM Corp., Armonk, NY, USA).

### 2.9. Ethics and Data Protection

The study was conducted within a quality-assurance framework using residual biological material collected during routine clinical care, with a waiver of individual informed consent in accordance with Norwegian regulations. The project was approved by the Regional Committee for Medical and Health Research Ethics, North Norway (REC Nord; protocol codes 2013/497/REK Nord and 2013/927/REK Nord, with subsequent amendments as applicable), and by the Data Protection Officer at the University Hospital of North Norway (UNN; reference 2016/2873).

## 3. Results

### 3.1. Study Population and Baseline Test Characteristics

A total of 123,674 co-test samples were initially identified. Of these, 116,715 (94.4%) had valid cytology results and 123,094 (99.5%) had valid HPV mRNA results. The final analytic cohort included 116,217 eligible co-test samples (94.0%) with both valid cytology and valid HPV mRNA results. Accordingly, 7457 samples (6.0%) were excluded because of unsatisfactory or indeterminate cytology and/or invalid HPV mRNA results resulting from a negative intrinsic sample control (ISC). The mean age at testing was 43.9 years (SD 14.4; range 20–93), reflecting a real-world cohort that included both routine screening samples and clinically indicated testing (e.g., follow-up/surveillance or diagnostic testing), including women outside the usual screening age range. During follow-up, 5089 women (4.4%) were diagnosed with CIN2+, including 1774 (1.5%) with CIN3+ and 126 (0.1%) with cervical cancer. At baseline, HPV mRNA positivity was observed in 4564 of 116,217 samples (3.9%). Among women who subsequently developed CIN2+, CIN3+, and cervical cancer, baseline HPV mRNA positivity was present in 47.4%, 62.1%, and 65.9%, respectively (Table 1).

### 3.2. Distribution of Histological Outcomes by Baseline HPV mRNA and Cytology

Baseline cytology was reported as NILM in 102,077 samples (87.8%) and as ASC-US+ in 14,140 samples (12.2%). The proportion of ASC-US+ cytology increased markedly with increasing disease severity, from 9.5% among women with no biopsy or <CIN2 to 71.4% among those with CIN2+, 78.0% among those with CIN3+, and 77.8% among women with cervical cancer (Table 1). High-grade cytology (ASC-H+), which accounted for 2.3% of all samples, represented a disproportionately large share of severe outcomes, comprising 32.6% of CIN2+, 49.7% of CIN3+, and 67.5% of cervical cancer cases.

When HPV mRNA and cytology results were combined, the majority of samples were double negative (mRNA−/NILM; 100,790 samples, 86.7%). Although absolute risk was lowest in this group, it nonetheless included 1025 CIN2+ cases (20.1% of all CIN2+), 224 CIN3+ cases (12.6% of all CIN3+), and 21 cervical cancers (16.7% of all cancers). In contrast, HPV mRNA positivity with NILM cytology was uncommon (1287 samples, 1.1%) but showed marked enrichment for high-grade disease, accounting for 431 CIN2+ cases (8.5%), 167 CIN3+ cases (9.4%), and 7 cervical cancers (5.6%). The highest-risk group consisted of women who were double positive (mRNA+/ASC-US+; 3277 samples, 2.8%), which accounted for 1979 CIN2+ cases (38.9% of all CIN2+), 934 CIN3+ cases (52.6% of all CIN3+), and 76 cervical cancers (60.3% of all cancers) (Table 1).

### 3.3. Absolute Risk of CIN2+, CIN3+, and Cervical Cancer by Co-Test Category

Absolute risks of CIN2+, CIN3+, and cervical cancer differed markedly across the four baseline co-test categories, forming a pronounced and clinically meaningful risk gradient (Table 2). Overall differences across co-test categories were statistically significant for all three endpoints (Pearson chi-square, all *p* < 0.001), although comparisons for cervical cancer should be interpreted cautiously because of sparse counts in some smaller subgroups.

**Figure 1:**
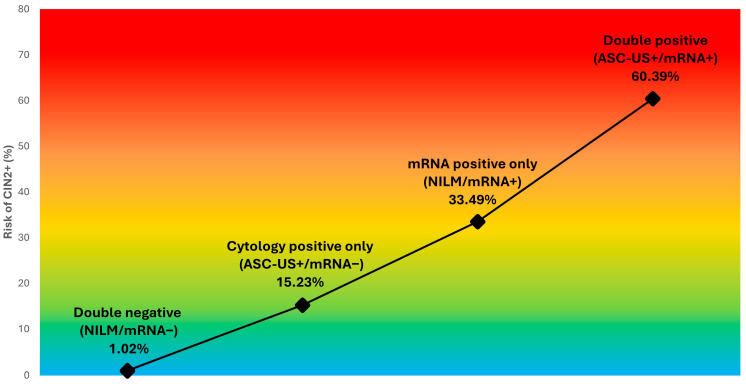
CIN2+ risk gradient across baseline co-test categories defined by cervical cytology and HPV mRNA status. The figure illustrates the absolute risk of CIN2+ during follow-up across four baseline co-test categories among eligible samples (N = 116,217): double negative (NILM/mRNA−; 1.02%), cytology-positive only (ASC-US+/mRNA−; 15.23%), mRNA-positive only (NILM/mRNA+; 33.49%), and double positive (ASC-US+/mRNA+; 60.39%). Absolute risks represent the proportion of women diagnosed with CIN2+ within each baseline co-test category during follow-up.

Women with double-negative results (NILM/mRNA−; *n* = 100,790, 86.7%) had very low absolute risks, with CIN2+ detected in 1.02% (1025/100,790; 95% CI 0.96–1.08), CIN3+ in 0.22% (224/100,790; 95% CI 0.20–0.25), and cervical cancer in 0.02% (21/100,790; 95% CI 0.01–0.03). Among women with abnormal cytology but negative HPV mRNA (ASC-US+/mRNA−; *n* = 10,863, 9.3%), risks increased substantially to 15.2% for CIN2+ (1654/10,863; 95% CI 14.56–15.91), 4.1% for CIN3+ (449/10,863; 95% CI 3.77–4.52), and 0.20% for cervical cancer (22/10,863; 95% CI 0.13–0.31) (Table 2; Figure 1).

Women with normal cytology but positive HPV mRNA (NILM/mRNA+; *n* = 1287, 1.1%) exhibited markedly elevated risks despite normal cytology, with CIN2+ observed in 33.5% (431/1287; 95% CI 30.96–36.11), CIN3+ in 13.0% (167/1287; 95% CI 11.25–14.92), and cervical cancer in 0.5% (7/1287; 95% CI 0.26–1.12). Compared with women with NILM/mRNA−, women with NILM/mRNA+ had substantially higher risks of CIN2+ (RR 32.9, 95% CI 29.85–36.33; risk difference 32.5 percentage points, 95% CI 29.89–35.05), CIN3+ (RR 58.39, 95% CI 48.15–70.79; risk difference 12.8 percentage points, 95% CI 10.92–14.59), and cervical cancer (RR 26.10, 95% CI 11.12–61.30; risk difference 0.52 percentage points, 95% CI 0.12–0.92).

The highest risks were observed among women with double-positive results (ASC-US+/mRNA+; *n* = 3277, 2.8%), in whom CIN2+, CIN3+, and cervical cancer occurred in 60.4% (1979/3277; 95% CI 58.70–62.05), 28.5% (934/3277; 95% CI 26.98–30.07), and 2.3% (76/3277; 95% CI 1.86–2.89), respectively (Table 2; Figure 1). Within the ASC-US+ cytology group, HPV mRNA positivity was associated with higher risks than HPV mRNA negativity for CIN2+ (RR 3.97, 95% CI 3.76–4.18; risk difference 45.2 percentage points, 95% CI 43.36–46.97), CIN3+ (RR 6.90, 95% CI 6.20–7.66; risk difference 24.4 percentage points, 95% CI 22.78–25.96), and cervical cancer (RR 11.45, 95% CI 7.14–18.38; risk difference 2.12 percentage points, 95% CI 1.59–2.64).

Across all eligible co-test samples (*n* = 116,217), cumulative risks were 4.38% for CIN2+, 1.53% for CIN3+, and 0.11% for cervical cancer.

### 3.4. Triage Performance of HPV mRNA Within Cytology Strata

#### 3.4.1. Risk Stratification Within NILM Cytology

Within women with NILM cytology (*n* = 102,077), baseline HPV mRNA positivity was uncommon (1287/102,077; 1.3%) but identified a markedly higher-risk subgroup with substantially increased risks across all endpoints (Table 3). Differences between NILM/mRNA− and NILM/mRNA+ were statistically significant for CIN2+, CIN3+, and cervical cancer (all *p* < 0.001; cervical cancer interpreted cautiously because of low event counts).

Among women who were mRNA-negative (*n* = 100,790), absolute risks were low, with CIN2+ in 1.02% (1025/100,790; 95% CI 0.96–1.08), CIN3+ in 0.22% (224/100,790; 95% CI 0.20–0.25), and cervical cancer in 0.02% (21/100,790; 95% CI 0.01–0.03). In contrast, among mRNA-positive women (*n* = 1287), risks increased substantially to 33.5% for CIN2+ (431/1287; 95% CI 30.96–36.11), 13.0% for CIN3+ (167/1287; 95% CI 11.25–14.92), and 0.54% for cervical cancer (7/1287; 95% CI 0.26–1.12).

Compared with NILM/mRNA−, the NILM/mRNA+ category was associated with markedly elevated risk, with risk ratios of 32.9 (95% CI 29.85–36.33) for CIN2+, 58.4 (95% CI 48.15–70.79) for CIN3+, and 26.1 (95% CI 11.12–61.30) for cervical cancer. The corresponding absolute risk differences were 32.5 percentage points (95% CI 29.89–35.05) for CIN2+, 12.75 percentage points (95% CI 10.92–14.59) for CIN3+, and 0.52 percentage points (95% CI 0.12–0.92) for cervical cancer.

Although the mRNA-positive subgroup represented only 1.3% of NILM samples, it accounted for 29.6% of CIN2+ cases within NILM (431/1456), 42.7% of CIN3+ cases (167/391), and 25.0% of cervical cancers (7/28). In practical terms, the absolute risk of cervical cancer following a NILM/mRNA+ result was 0.54% (approximately 1 in 184), compared with 0.021% (approximately 1 in 4800) following a NILM/mRNA− result.

#### 3.4.2. Risk Stratification Within ASC-US+ Cytology

Within women with ASC-US+ cytology (*n* = 14,140), baseline HPV mRNA positivity was common (3277/14,140; 23.2%) and clearly stratified risk across all endpoints (Table 4). Differences between ASC-US+/mRNA− and ASC-US+/mRNA+ were statistically significant for CIN2+, CIN3+, and cervical cancer (all *p* < 0.001), with interpretation of the cancer endpoint based on fewer events than the CIN endpoints.

Among women who were mRNA-negative (*n* = 10,863), absolute risks were 15.2% for CIN2+ (1654/10,863; 95% CI 14.56–15.91), 4.1% for CIN3+ (449/10,863; 95% CI 3.77–4.52), and 0.20% for cervical cancer (22/10,863; 95% CI 0.13–0.31). In contrast, among mRNA-positive women (*n* = 3277), risks increased markedly to 60.4% for CIN2+ (1979/3277; 95% CI 58.70–62.05), 28.5% for CIN3+ (934/3277; 95% CI 26.98–30.07), and 2.3% for cervical cancer (76/3277; 95% CI 1.86–2.89).

Compared with ASC-US+/mRNA− women, those who were ASC-US+/mRNA+ had substantially elevated risks, with risk ratios of 4.0 (95% CI 3.76–4.18) for CIN2+, 6.9 (95% CI 6.20–7.66) for CIN3+, and 11.5 (95% CI 7.14–18.38) for cervical cancer. The corresponding absolute risk differences were 45.2 percentage points (95% CI 43.36–46.97) for CIN2+, 24.4 percentage points (95% CI 22.78–25.96) for CIN3+, and 2.1 percentage points (95% CI 1.59–2.64) for cervical cancer.

Although the mRNA-positive subgroup comprised only 23.2% of ASC-US+ samples, it accounted for the majority of adverse outcomes within this cytology category, including 54.5% of CIN2+ cases (1979/3633), 67.5% of CIN3+ cases (934/1383), and 77.6% of cervical cancers (76/98).

### 3.5. Genotype-Specific HPV mRNA Positivity and Risk

Genotype-specific HPV mRNA positivity demonstrated marked differences in risk across the three targeted HPV types (Table 5), with a clear gradient for CIN2+ and CIN3+ outcomes (HPV16 > HPV18 > HPV45).

Among samples positive for HPV16 mRNA (*n* = 2406), 66.8% developed CIN2+ (1608/2406), 35.3% developed CIN3+ (848/2406), and 2.2% developed cervical cancer (54/2406). HPV18 mRNA positivity (*n* = 1175) was also associated with substantial risk, with CIN2+ observed in 51.9% (610/1175), CIN3+ in 21.3% (250/1175), and cervical cancer in 2.0% (23/1175). In contrast, HPV45 mRNA positivity (*n* = 1474) identified a comparatively lower-risk subgroup, with CIN2+ in 32.8% (483/1474), CIN3+ in 9.3% (137/1474), and cervical cancer in 0.6% (9/1474).

These marker-specific estimates support clinically meaningful risk stratification, particularly for CIN2+ and CIN3+, and indicate that HPV16 mRNA positivity is associated with the highest subsequent risk, whereas HPV45 mRNA positivity is associated with substantially lower risk. The difference between HPV16 and HPV18 was more pronounced for CIN2+ and CIN3+ than for cervical cancer, for which the absolute proportions were similar (2.2% vs. 2.0%) and event counts were lower.

Because genotype-specific mRNA markers are not mutually exclusive and co-expression may occur within the same sample, Table 5 is presented primarily as a descriptive, marker-specific analysis. Formal between-group statistical comparisons were not emphasized for Table 5 because the marker groups overlap and are therefore not independent. To address this, we performed an additional analysis using mutually exclusive genotype-combination groups (Table 6), which allowed inferential comparison of outcome distributions across genotype combinations.

In the genotype-combination analysis restricted to HPV mRNA-positive samples, mutually exclusive HPV16/18/45 mRNA combinations showed marked differences in subsequent histological outcomes (Table 6). Overall differences across genotype-combination groups were statistically significant for CIN2+ (Pearson χ^2^ = 484.9, df = 6, *p* < 0.001) and CIN3+ (Pearson χ^2^ = 381.6, df = 6, *p* < 0.001). For cervical cancer, the overall comparison was also statistically significant (Pearson χ^2^ = 19.2, df = 6, *p* = 0.004), but this endpoint should be interpreted cautiously because of sparse cell counts in several multi-genotype categories.

HPV16-containing combinations had the highest risks overall. Among single-genotype positive samples, HPV16-only positivity was associated with substantially higher risks of CIN2+ and CIN3+ (66.3% and 35.1%, respectively) than HPV18-only positivity (49.7% and 20.2%) and HPV45-only positivity (28.5% and 6.5%). Double-positive combinations including HPV16 (HPV16 + 18 and HPV16 + 45) also showed high proportions of CIN2+ (76.8% and 74.7%) and CIN3+ (39.0% and 40.5%), whereas HPV18 + 45 had lower risks (51.0% and 13.5%), closer to HPV18-only than to HPV16-containing combinations. Cervical cancer was observed most often in the HPV16-only (2.5%) and HPV18-only (2.3%) groups and less often in the HPV45-only group (0.7%); no cancer cases were observed in several multi-genotype groups, but these estimates are imprecise because of small sample sizes. Overall, these findings indicate that risk was driven mainly by genotype composition—particularly the presence of HPV16—rather than by the number of positive genotype markers alone.

## 4. Discussion

### 4.1. Principal Findings and Clinical Risk Stratification

In this large, real-world co-testing cohort, the combination of cervical cytology with a genotype-specific HPV mRNA assay targeting HPV16, HPV18, and HPV45 (PreTect SEE) produced a pronounced and clinically intuitive risk gradient across all endpoints. The four baseline co-test categories separated women into groups ranging from very low risk (NILM/mRNA−) to extremely high risk (ASC-US+/mRNA+), with stepwise increases in absolute risk for CIN2+, CIN3+, and cervical cancer. These findings support the concept that HPV mRNA positivity, reflecting transcriptionally active infection, adds substantial prognostic information beyond cytology alone, while maintaining a low overall test positivity in the screened population. Importantly, these results do not challenge the role of HPV DNA testing as the primary screening modality but rather illustrate how genotype-specific HPV mRNA testing may complement cytology within existing screening and follow-up pathways.

### 4.2. HPV mRNA Testing in Women with Normal Cytology (NILM)

A key finding is the utility of HPV mRNA testing among women with NILM cytology. Although only 1.3% of NILM samples were mRNA positive, this small subgroup accounted for a disproportionate share of serious outcomes: 42.7% of CIN3+ lesions and 25.0% of cervical cancers occurring within NILM were observed among mRNA-positive women. Clinically, the risk contrast was substantial. The absolute risk of cervical cancer following a NILM/mRNA+ result was approximately 0.5% (about 1 in 184), compared with 0.02% (about 1 in 4800) after a NILM/mRNA− result. These findings suggest that HPV mRNA testing can meaningfully reduce residual cancer risk after a normal cytology result by identifying a small, actionable subgroup warranting closer evaluation, without substantially increasing referrals among women with NILM overall [25]. Although risk was very low in the double-negative (NILM/mRNA−) group, it was not zero. Residual CIN3+ and cervical cancer cases likely reflect, at least in part, lesions associated with HPV types not targeted by the 3-type HPV mRNA assay (HPV16/18/45), HPV-undetected tumors, and/or limitations related to sampling and routine clinical follow-up.

### 4.3. HPV mRNA as a Triage Tool Within ASC-US+ Cytology

Among women with ASC-US+ cytology, HPV mRNA further refined risk stratification by identifying a higher-risk subgroup with markedly elevated absolute risks, particularly for CIN3+ and cervical cancer. Although mRNA positivity was more common in this cytology category (23.2%), the mRNA-positive subgroup accounted for the majority of adverse outcomes within ASC-US+, including 77.6% of cervical cancers. This supports a triage role for HPV mRNA within equivocal or low-grade cytology, where it may help prioritize women most likely to benefit from immediate colposcopy while reducing unnecessary procedures among mRNA-negative women [26].

### 4.4. Genotype-Specific Risk and Biological Plausibility

Genotype-specific analyses further underscore the biological plausibility and clinical interpretability of the findings. HPV16 mRNA positivity was associated with the highest absolute risks and positive predictive values for CIN3+ and cervical cancer, followed by HPV18, whereas HPV45 mRNA positivity was associated with lower absolute risks. This hierarchy is consistent with established genotype-specific oncogenic potential and suggests that genotype-level mRNA information may be useful not only for risk stratification but also for communicating risk and tailoring follow-up intensity. This may be particularly relevant in the NILM triage setting, where absolute risks diverged markedly between mRNA-negative and mRNA-positive women [27].

Taken together, these results indicate that HPV mRNA-based co-testing provides strong and clinically interpretable risk stratification in routine practice. By identifying women at highest risk—especially those with normal cytology who would otherwise be reassured—this approach offers a pragmatic strategy to improve precision in cervical cancer screening without relying on complex risk models or substantially increasing referral burden [25].

### 4.5. Comparison with Prior Studies and Screening Strategies

Our findings extend and complement prior evidence demonstrating that combining HPV testing with cytology can yield strong, clinically interpretable risk stratification. In large routine-practice cohorts using broad HPV DNA assays, co-testing has consistently separated women into groups with very low risk after double-negative results and progressively higher risk when HPV and/or cytology is positive. These observations underpin recommendations for extended screening intervals after double-negative tests and intensified follow-up for discordant or double-positive results [28].

When compared with prior Norwegian studies using genotype-specific HPV mRNA assays, the pattern and magnitude of risk enrichment observed among cytology-negative, mRNA-positive women are consistent with a “small-positive, high-risk” profile. In quality-assurance cohorts, HPV mRNA positivity among women with normal cytology has been uncommon (typically around 2–3%) but associated with a high subsequent risk of CIN3+ during follow-up, indicating that transcriptional HPV activity can identify clinically relevant disease not captured by morphology alone [23]. In our cohort, the NILM/mRNA+ group was similarly small (1.3%) yet concentrated a substantial proportion of severe outcomes within NILM, underscoring the potential of HPV mRNA as an adjunct marker to reduce residual cancer risk after a normal cytology result.

Differences in absolute risks across studies are expected and likely reflect variation in study design and population composition. Several prior analyses were restricted to primary screening populations with explicit exclusions, such as prior abnormalities or narrow age ranges, whereas our real-world dataset includes routine clinical practice encompassing screening, follow-up, and symptom-driven testing across a broad age span. In primary-screening cohorts using HPV mRNA testing, mRNA negativity has been associated with low cumulative CIN3+ risk over multi-year follow-up, supporting the high reassurance value of a negative mRNA result, while also illustrating that absolute risk estimates depend on baseline risk and follow-up intensity [29].

Within ASC-US+ cytology, our findings align with previous triage literature showing that HPV mRNA testing can improve specificity and reduce unnecessary referrals compared with broad HPV DNA testing, while maintaining clinically important detection of higher-grade disease [17,18]. In large European evaluations—including head-to-head comparisons of mRNA assays with partial genotyping versus DNA-based tests in primary screening settings, as well as randomized screening trial data—HPV *E6/E7* mRNA testing has demonstrated competitive performance with the potential for fewer test positives and downstream assessments at comparable detection of CIN3+ [20,21]. Consistent with this, we observed that among women with ASC-US+ cytology, the majority of CIN3+ lesions and cervical cancers occurred in the mRNA-positive subgroup, supporting a triage role for HPV mRNA to prioritize colposcopy toward those at highest risk while allowing de-escalation among mRNA-negative women.

Our results are also broadly concordant with international experience using HPV mRNA testing platforms that detect *E6/E7* transcripts across multiple HPV types, which have generally demonstrated sensitivity comparable to HPV DNA testing with improved specificity for clinically relevant endpoints [17]. Although the assay evaluated here is genotype-specific (HPV16/18/45) rather than broad, the same conceptual advantage applies: detection of oncogene transcription helps distinguish infections more likely to be associated with high-grade disease from transient infections identified by DNA presence alone.

The genotype-specific hierarchy observed in our study—highest risks among HPV16 mRNA-positive women, followed by HPV18, with lower absolute risks for HPV45—is biologically plausible and consistent with established differences in oncogenic potential, as well as with prior Norwegian mRNA studies reporting high CIN3+ risk among HPV16/18/45 mRNA-positive women with initially normal cytology [29]. This supports the clinical interpretability of genotype-level mRNA results and suggests a practical framework in which HPV16 mRNA positivity signals the need for the most immediate work-up, while HPV18 and HPV45 positivity may warrant close follow-up integrated with cytology and prior screening history [27].

### 4.6. Implementation Considerations and Clinical Implications

From an implementation perspective, our data highlight a pragmatic use case: adding genotype-specific HPV mRNA testing to cytology can generate a steep risk gradient with a low positivity rate among NILM samples, potentially improving program sensitivity without a substantial increase in workload from cytology-negative referrals [23]. While quality-assurance studies suggest that testing a small fraction of cytology-normal samples that are HPV mRNA-positive could increase detection of high-grade disease, formal cost-effectiveness analyses will be needed to balance incremental detection against additional testing costs and downstream procedures [25]. Although WHO recommends HPV DNA testing as the preferred primary screening method, cytology-based screening and mixed transition models remain relevant in many healthcare systems, particularly where full implementation of HPV DNA-based screening is still in progress. In this context, our findings have broader relevance by showing that a targeted genotype-specific HPV mRNA assay can provide strong risk stratification with low positivity, which may support pragmatic implementation in cytology-based programs, triage pathways, and selected low-resource settings. This may be particularly useful when the goal is to improve detection of clinically important lesions while limiting unnecessary follow-up and treatment. However, the optimal role of HPV mRNA testing will depend on local screening infrastructure, assay availability, costs, and follow-up capacity, and should be evaluated in setting-specific implementation and cost-effectiveness studies.

### 4.7. Strengths and Limitations

This study has several important strengths. It is based on a large, real-world cohort from routine clinical practice in a population-based screening setting, with long-term passive follow-up through a regional pathology information system. This enabled linkage of baseline co-test results to subsequent histological outcomes captured in routine care and allowed estimation of absolute risks that are directly clinically interpretable. In addition, the availability of genotype-specific HPV mRNA results (HPV16, HPV18, and HPV45) allowed clinically relevant risk stratification beyond binary test positivity, including exploratory analyses of mutually exclusive genotype-combination groups among HPV mRNA-positive samples.

Several limitations should also be considered when interpreting the findings and when comparing results across studies. First, passive outcome ascertainment through routine clinical care may introduce verification bias, as women with abnormal baseline test results (abnormal cytology and/or HPV mRNA-positive results) are more likely to undergo prompt colposcopy and biopsy than women with double-negative baseline results, who are typically returned to routine screening intervals. This may inflate risk estimates in test-positive groups and affect comparisons with double-negative groups. However, this follow-up pattern reflects real-world screening practice, and the cohort was followed through multiple screening rounds with histological outcomes captured through December 2025, allowing some lesions missed at baseline to be detected later during follow-up. In addition, the gradual implementation of HPV DNA-based primary screening from 2019 may have reduced the risk of undetected CIN2+ and cervical cancer during later follow-up.

Second, the unit of analysis was the co-test sample rather than unique individuals; repeated testing in follow-up or surveillance settings may therefore inflate event counts relative to cohorts restricted to primary screening only. Third, although outcomes were captured through a comprehensive regional pathology system, procedures performed outside the catchment area may not have been fully ascertained, which may have led to underestimation of outcomes. Nevertheless, deterministic linkage within the laboratory information system supports high completeness for locally managed care pathways.

A further limitation is that HPV DNA test results were not available for the full cohort and were therefore not included in the present analyses. During most of the study period, the Norwegian screening program was cytology-based, and primary HPV DNA testing was implemented gradually from 2019. Consequently, most women in this cohort had cytology and 3-type HPV mRNA co-testing only. In addition, the HPV DNA assay used in screening (Roche cobas 4800) provided only partial genotyping (HPV16, HPV18, and pooled other HPV types), which did not allow separate identification of HPV45. HPV DNA results were therefore available only in a selected subgroup and were not directly comparable across the full cohort. Accordingly, the clinical value of PreTect SEE should be interpreted as an adjunct to cytology in this historical screening context, not as a stand-alone alternative to contemporary HPV DNA-based primary screening.

Additional limitations relate to statistical interpretation. The analyses were primarily descriptive and based on cross-tabulations and cumulative risks at the co-test sample level; therefore, the results support risk stratification rather than causal inference. Some reported outcome categories (CIN2+, CIN3+, and cervical cancer) are nested, and corresponding distributions were presented descriptively. For cervical cancer and for some genotype-combination subgroups, event counts were small, resulting in less precise estimates and requiring cautious interpretation of *p*-values and between-group comparisons.

A further limitation is that this study did not include a formal health-economic evaluation. Although the observed risk stratification may support clinical utility, implementation in organized screening programs also depends on cost-effectiveness, resource use, referral burden, and program-level feasibility, which were outside the scope of the present analyses.

Finally, temporal changes in screening policies, HPV vaccination uptake, and referral practices during the study period may have influenced absolute risk estimates. Future analyses should address these factors using stratified analyses and/or calendar-period-specific approaches.

### 4.8. Overall Interpretation

Overall, viewed alongside prior Norwegian and international evidence, our findings support the role of HPV mRNA—particularly genotype-specific *E6/E7* detection—as a clinically meaningful adjunct to cervical cytology. This approach provides strong and clinically interpretable risk stratification, identifies a small but high-risk subgroup among women with normal cytology, and refines triage among ASC-US+ by concentrating CIN3+ and cervical cancer risk in HPV mRNA-positive women [17].

## 5. Conclusions

In this large real-world cohort of co-test samples from routine cervical screening, follow-up, and clinically indicated testing in Northern Norway, co-testing with cervical cytology and a genotype-specific HPV mRNA assay targeting HPV16, HPV18, and HPV45 (PreTect SEE) provided strong and clinically intuitive risk stratification for CIN2+, CIN3+, and cervical cancer. The four baseline co-test categories formed a clear stepwise risk gradient, ranging from very low risk among double-negative samples to extremely high risk among double-positive samples. HPV mRNA testing added particular value among women with NILM cytology by identifying a small subgroup (1.3%) with markedly elevated risk and capturing a substantial proportion of CIN3+ lesions and cervical cancers occurring after an otherwise reassuring cytology result. Among women with ASC-US+ cytology, HPV mRNA further refined risk stratification by concentrating most CIN3+ and cervical cancer outcomes in the mRNA-positive subgroup, supporting a triage role to prioritize colposcopy while potentially reducing unnecessary procedures among mRNA-negative women.

Genotype-specific mRNA results were clinically interpretable and biologically plausible, with the highest risks observed in HPV16-containing patterns, followed by HPV18, and lower risks for HPV45. Taken together, these findings support genotype-specific HPV mRNA testing as a clinically meaningful adjunct to cytology for risk stratification in routine practice, particularly for identifying higher-risk women despite normal cytology. However, the analyses were descriptive and performed at the co-test sample level in a mixed clinical population; therefore, the findings should not be interpreted as causal and may not be directly generalizable to strictly defined primary-screening cohorts. Future studies should address optimal management thresholds, referral strategies, and cost-effectiveness in contemporary vaccinated populations and within current HPV DNA-based screening algorithms.

## Figures and Tables

**Table 1:** Distribution of histological outcomes according to baseline HPV mRNA status and cervical cytology among eligible co-test samples.

Characteristic	All Samples	No Biopsy or <CIN2	CIN2+	CIN3+	Cervical Cancer
Total	116,217 (100%)	111,128 (100%)	5089 (100%)	1774 (100%)	126 (100%)
Baseline HPV mRNA (PreTect SEE)					
Negative	111,653 (96.1%)	108,974 (98.1%)	2679 (52.6%)	673 (37.9%)	43 (34.1%)
Positive	4564 (3.9%)	2154 (1.9%)	2410 (47.4%)	1101 (62.1%)	83 (65.9%)
Cervical cytology					
NILM	102,077 (87.8%)	100,621 (90.5%)	1456 (28.6%)	391 (22.0%)	28 (22.2%)
ASC-US+	14,140 (12.2%)	10,507 (9.5%)	3633 (71.4%)	1383 (78.0%)	98 (77.8%)
ASC-H+ (subset of ASC-US+)	2716 (2.3%)	1059 (1.0%)	1657 (32.6%)	881 (49.7%)	85 (67.5%)
Baseline Cervical cytology/ HPV mRNA					
NILM/mRNA−	100,790 (86.7%)	99,765 (89.8%)	1025 (20.1%)	224 (12.6%)	21 (16.7%)
ASC-US+/mRNA−	10,863 (9.3%)	9209 (8.3%)	1654 (32.5%)	449 (25.3%)	22 (17.5%)
NILM/mRNA+	1287 (1.1%)	856 (0.8%)	431 (8.5%)	167 (9.4%)	7 (5.6%)
ASC-US+/mRNA+	3277 (2.8%)	1298 (1.2%)	1979 (38.9%)	934 (52.6%)	76 (60.3%)

Abbreviations: NILM, negative for intraepithelial lesion or malignancy; ASC-US+, atypical squamous cells of undetermined significance or more severe abnormalities; ASC-H+, atypical squamous cells, cannot exclude high-grade squamous intraepithelial lesion (HSIL), or more severe abnormalities; CIN, cervical intraepithelial neoplasia. CIN3+ includes CIN3, adenocarcinoma in situ (AIS), and cervical cancer. Cervical cancer includes invasive squamous cell carcinoma and invasive adenocarcinoma. HPV mRNA refers to HPV *E6/E7* mRNA targeting HPV16, HPV18, and HPV45 (PreTect SEE). Outcomes were assessed over follow-up.

**Table 2:** Absolute risks of CIN2+, CIN3+, and cervical cancer according to baseline co-test category among eligible co-test samples (N = 116,217).

Co-Test Category	N	CIN2+ *n* (%)	CIN3+ *n* (%)	Cervical Cancer *n* (%)
NILM/mRNA−	100,790	1025 (1.02)	224 (0.22)	21 (0.02)
ASC-US+/mRNA−	10,863	1654 (15.23)	449 (4.13)	22 (0.20)
NILM/mRNA+	1287	431 (33.49)	167 (12.98)	7 (0.54)
ASC-US+/mRNA+	3277	1979 (60.39)	934 (28.50)	76 (2.32)
Total	116,217	5089 (4.38)	1774 (1.53)	126 (0.11)

Abbreviations: NILM, negative for intraepithelial lesion or malignancy; ASC-US+, atypical squamous cells of undetermined significance or more severe abnormalities; mRNA−/mRNA+, negative/positive HPV *E6/E7* mRNA test targeting HPV16, HPV18, and HPV45 (PreTect SEE); CIN2+, cervical intraepithelial neoplasia grade 2 or worse; CIN3+, cervical intraepithelial neoplasia grade 3 or worse, including adenocarcinoma in situ (AIS) and cervical cancer; cervical cancer, invasive squamous cell carcinoma or adenocarcinoma.

**Table 3:** Triage performance of HPV mRNA within NILM cytology (N = 102,077).

NILM Cytology	N	CIN2+ n (%)	CIN3+ n (%)	Cervical Cancer n (%)	RR (mRNA+ vs. mRNA−)
mRNA−	100,790	1025 (1.0)	224 (0.2)	21 (0.02)	ref
mRNA+	1287	431 (33.5)	167 (13.0)	7 (0.54)	32.9/58.4/26.1

Abbreviations: NILM, negative for intraepithelial lesion or malignancy; mRNA−/mRNA+, negative/positive HPV *E6/E7* mRNA test targeting HPV16, HPV18, and HPV45 (PreTect SEE); CIN2+, cervical intraepithelial neoplasia grade 2 or worse; CIN3+, cervical intraepithelial neoplasia grade 3 or worse, including adenocarcinoma in situ (AIS) and cervical cancer; cervical cancer, invasive squamous cell carcinoma or invasive adenocarcinoma; RR, risk ratio; Ref, reference group.

**Table 4:** Triage performance of HPV mRNA within ASC-US+ cytology (N = 14,140).

ASC-US+ Cytology	N	CIN2+ *n* (%)	CIN3+ *n* (%)	Cervical Cancer *n* (%)	RR (mRNA+ vs. mRNA−)
mRNA−	10,863	1654 (15.2)	449 (4.1)	22 (0.2)	Ref
mRNA+	3277	1979 (60.4)	934 (28.5)	76 (2.3)	4.0/6.9/11.5

Abbreviations: ASC-US+, atypical squamous cells of undetermined significance or more severe abnormalities; mRNA−/mRNA+, negative/positive HPV *E6/E7* mRNA test targeting HPV16, HPV18, and HPV45 (PreTect SEE); CIN2+, cervical intraepithelial neoplasia grade 2 or worse; CIN3+, cervical intraepithelial neoplasia grade 3 or worse, including adenocarcinoma in situ (AIS) and cervical cancer; cervical cancer, invasive squamous cell carcinoma or invasive adenocarcinoma; RR, risk ratio; Ref, reference group.

**Table 5:** Histological outcomes according to genotype-specific HPV mRNA positivity (markers not mutually exclusive).

Genotype Marker	N Positive	CIN2+, *n* (%)	CIN3+, *n* (%)	Cervical Cancer, *n* (%)
HPV16 mRNA+	2406	1608 (66.8)	848 (35.3)	54 (2.2)
HPV18 mRNA+	1175	610 (51.9)	250 (21.3)	23 (2.0)
HPV45 mRNA+	1474	483 (32.8)	137 (9.3)	9 (0.6)

Abbreviations: HPV, human papillomavirus; mRNA+, positive HPV *E6/E7* mRNA test for the specified genotype (HPV16, HPV18, or HPV45; PreTect SEE); CIN2+, cervical intraepithelial neoplasia grade 2 or worse; CIN3+, cervical intraepithelial neoplasia grade 3 or worse, including adenocarcinoma in situ (AIS) and cervical cancer; cervical cancer, invasive squamous cell carcinoma or invasive adenocarcinoma. Note: Percentages are calculated within genotype-specific HPV mRNA–positive samples. Genotype markers are not mutually exclusive.

**Table 6:** Histological outcomes according to mutually exclusive HPV mRNA genotype combinations (HPV16/18/45) among HPV mRNA-positive samples.

HPV mRNA	N Positive	CIN2+, *n* (%)	CIN3+, *n* (%)	Cervical Cancer, *n* (%)
HPV16 only	2148	1424 (66.3)	753 (35.1)	53 (2.5)
HPV18 only	919	457 (49.7)	186 (20.2)	21 (2.3)
HPV45 only	1222	348 (28.5)	80 (6.5)	8 (0.7)
HPV16 + 18	82	63 (76.8)	32 (39.0)	1 (1.2)
HPV16 + 45	79	59 (74.7)	32 (40.5)	0 (0.0)
HPV18 + 45	96	49 (51.0)	13 (13.5)	0 (0.0)
HPV16 + 18 + 45	18	10 (55.6)	5 (27.8)	0 (0.0)

Abbreviations: HPV, human papillomavirus; mRNA+, positive HPV *E6/E7* mRNA test for the specified genotype (HPV16, HPV18, or HPV45; PreTect SEE); CIN2+, cervical intraepithelial neoplasia grade 2 or worse; CIN3+, cervical intraepithelial neoplasia grade 3 or worse, including adenocarcinoma in situ (AIS) and cervical cancer; cervical cancer, invasive squamous cell carcinoma or invasive adenocarcinoma. Percentages are calculated within each mutually exclusive genotype-combination group. Analysis restricted to samples with valid cytology and valid HPV mRNA results. Small cell counts in some multi-genotype groups (especially triple-positive and cancer outcomes) require cautious interpretation.

## Data Availability

De-identified analysis datasets and statistical code supporting the findings of this study are available from the corresponding author upon reasonable request. The underlying individual-level data cannot be shared publicly due to privacy, ethical, and legal restrictions under Norwegian data protection regulations and institutional policies. Aggregate results are provided within the article.

## Abbreviations

The following abbreviations are used in this manuscript:

AIS	Adenocarcinoma in situ
ASC-H	Atypical squamous cells—cannot exclude HSIL
ASC-H+	Atypical squamous cells—cannot exclude HSIL or worse
ASC-US	Atypical squamous cells of undetermined significance
ASC-US+	Atypical squamous cells of undetermined significance or worse
CI	Confidence interval
CIN2	Cervical intraepithelial neoplasia grade 2
CIN2+	Cervical intraepithelial neoplasia grade 2 or worse
CIN3	Cervical intraepithelial neoplasia grade 3
CIN3+	Cervical intraepithelial neoplasia grade 3 or worse
DNA	Deoxyribonucleic acid
E6/E7	Early genes E6 and E7
HPV	Human papillomavirus
HSIL	High-grade squamous intraepithelial lesion
ISC	Intrinsic sample control
LBC	Liquid-based cytology
LIS	Laboratory information system
LSIL	Low-grade squamous intraepithelial lesion
mRNA	Messenger RNA
NASBA	Nucleic acid sequence-based amplification
NILM	Negative for intraepithelial lesion or malignancy
NPV	Negative predictive value
PPV	Positive predictive value
PreTect SEE	Genotype-specific HPV *E6/E7* mRNA test (HPV16/18/45)
REC	Regional Committee for Medical and Health Research Ethics (REC Nord)
RR	Risk ratio
SCC	Squamous cell carcinoma
ThinPrep	ThinPrep liquid-based cytology system
UNN	University Hospital of North Norway
